# Human Papillomavirus (HPV) Self-Sampling among Never-and Under-Screened Indigenous Māori, Pacific and Asian Women in Aotearoa New Zealand: A Feasibility Study

**DOI:** 10.3390/ijerph181910050

**Published:** 2021-09-24

**Authors:** Collette Bromhead, Helen Wihongi, Susan M. Sherman, Sue Crengle, Jane Grant, Georgina Martin, Anna Maxwell, Georgina McPherson, ‘Aivi Puloka, Susan Reid, Nina Scott, Karen Bartholomew

**Affiliations:** 1 School of Health Sciences, Massey University, Wellington 6021, New Zealand; c.bromhead@massey.ac.nz; 2 Ngāti Porou, Ngāpuhi, Te Whānau a Apanui, Ngāti Hine, Waitematā District Health Board and Auckland DHB, Private Bag 93-503, Takapuna, Auckland 0740, New Zealand; Helen.Wihongi@waitematadhb.govt.nz; 3 School of Psychology, Keele University, Keele ST5 5BG, UK; s.m.sherman@keele.ac.uk; 4 Waitaha, Kāti Mamoe and Kāi Tahu, Department of Preventive and Social Medicine, University of Otago, Dunedin 9016, New Zealand; sue.crengle@otago.ac.nz; 5 Waitematā District Health Board and Auckland DHB, Private Bag 93-503, Takapuna, Auckland 0740, New Zealand; jane.grant@waitematadhb.govt.nz (J.G.); Anna.maxwell@waitematadhb.govt.nz (A.M.); 6 Te Rarawa, Te Aupōuri, Ngāpuhi, Wai Research, Te Whānau O Waipareira. Level 1, 6-8 Pioneer Street, Henderson, Waitakere City, Auckland 0605, New Zealand; wairesearch@waiwhanau.com; 7 Makea Ngāti Kao, Ngāti Tane, Waitematā District Health Board, Auckland 0740, New Zealand; georgina.mcpherson@waitematadhb.govt.nz; 8 The Fono Health & Social Services, Henderson, Auckland 0612, New Zealand; aivippuloka@gmail.com; 9 Te Rarawa, Health Literacy New Zealand Limited, Auckland 1347, New Zealand; sreid@healthliteracy.co.nz; 10 Ngāpuhi, Ngāti Whātua, Waikato, Waikato District Health Board, Hamilton 3240, New Zealand; nscott.waikato@gmail.com

**Keywords:** indigenous health research, equity, cervical cancer screening, HPV, self-sampling, Kaupapa Māori Research

## Abstract

In Aotearoa, New Zealand, the majority of cervical cancer cases occur in women who have never been screened or are under-screened. Wāhine Māori, Pacific and Asian women have the lowest rate of cervical screening. Self-sampling for human papillomavirus (HPV-SS) has been shown to increase participation in cervical cancer screening. A whole-of-system approach, driven by evidence in the most effective delivery of HPV-SS, is required to mitigate further widening of the avoidable gap in cervical screening access and outcomes between groups of women in Aotearoa. This single-arm feasibility and acceptability study of HPV self-sampling invited never- and under-screened (≥5 years overdue) 30–69-year-old women from general practices in Auckland, Aotearoa. Eligible women were identified by data matching between the National Cervical Programme (NCSP) Register and practice data. Focus groups were additionally held with eligible wāhine Māori, Asian and Pacific women to co-design new patient information materials. Questionnaires on HPV knowledge and post-test experience were offered to women. Our follow-up protocols included shared decision-making principles, and we committed to follow-up ≥90% of women who tested positive for HPV. Data matching identified 366 eligible never- and under-screened wāhine Māori, Pacific and Asian women in participating practices. We were only able to contact 114 women, and 17, during the discussion, were found to be ineligible. Identifying and contacting women overdue for a cervical screen was resource-intensive, with a high rate of un-contactability despite multiple attempts. We found the best uptake of self-sampling was at focus groups. Of the total 84 HPV-SS tests, there were five positive results (6%), including one participant with HPV18 who was found to have a cervical Adenocarcinoma at colposcopy. In our feasibility study, self-sampling was acceptable and effective at detecting HPV and preventing cervical cancer in under-screened urban wāhine Māori, Pacific and Asian women in Aotearoa. This is the first report of cervical Adenocarcinoma (Grade 1B) as a result of an HPV-18 positive self-sample in Aotearoa. We co-designed new patient information materials taking a health literacy and ethnicity-specific approach. This work provides policy-relevant information to the NCSP on the resources required to implement an effective HPV self-sampling programme to improve equity in national cervical cancer screening.

## 1. Background

The current National Cervical Screening Programme (NCSP) in Aotearoa invites women every three years between the ages of 25–69 and uses liquid-based cytology as the primary test. The NCSP has been successful at reducing cervical cancer incidence and mortality. As a result, cervical cancer is a relatively rare cancer from a total population perspective [1,2]. A recent Invasive Cervical Cancer Audit confirmed that most women in Aotearoa who develop cervical cancer are either unscreened or under-screened [3,4,5]. The groups least served in Aotearoa by the NCSP were Indigenous wāhine (Māori women), as well as Pacific and Asian women. Screening coverage for all groups was well below the 80% target, with 2019 three-year rates 12–21% less than other ethnicities (mostly NZ European). Despite multiple evidence-based strategies to improve cervical screening coverage, none have yet made a significant difference across all ethnicities [6]. 

In 2018 the World Health Organization (WHO) Director-General issued a global call for action to eliminate cervical cancer [7]. Elimination is considered possible because cervical cancer is a preventable cancer through the twin prevention strategies of HPV vaccination and cervical screening with a primary HPV test (followed by treatment of precancerous lesions) [8,9]. Aotearoa introduced HPV vaccination for females in 2008 and has completed significant policy and planning work to implement HPV primary screening [10], intended for 2018 but now delayed until 2023 [11].

HPV primary screening opens up the possibility of HPV self-sampling, which is comparable to clinician cervical sampling [12,13,14] and can be conducted at the clinic or outside the health system, bypassing the commonly cited barriers [15]. HPV self-sampling has increased screening participation in never- or under-screened populations in a variety of countries, including recent trials in Aotearoa [16,17,18,19,20]. We conducted a feasibility study between 2016 to 2017 to trial novel wrap-around, culturally safe HPV self-sampling and follow-up processes for never- or under-screened wāhine Māori, Pacific and Asian women. Our study was based in an urban primary care setting and reflected a possible model for implementation in the NCSP, which proposes to offer self-sampling as part of HPV primary screening in 2023 [21].

The study was also designed to optimise women’s experience of HPV self-sampling via a series of focus groups, including knowledge and acceptability questionnaires. While using a co-design and health literacy approach, our study aimed to evaluate the acceptability, uptake and cultural appropriateness of an HPV self-sampling experience amongst Indigenous wāhine Māori, Pacific and Asian women. We also sought to obtain information on the resources required to achieve 90% follow-up of HPV-positive women.

## 2. Methods

### 2.1. Indigenous Māori Community Engagement, Involvement and Leadership

The study focused on a preventable health condition with substantial inequities in outcomes for wāhine Māori. Planning for the introduction of novel technology such as self-sampling was informed by early dissemination of He Pikinga Waiora, a Māori implementation science framework [22]. The topic was identified by Hei Āhuru Mowai, the Māori Cancer Leadership Network, and included in the national and the District Health Boards Māori Health Plans. The study team included Māori researchers, advisors and community members (HW, SC, NS, GM, GMcP, SR, AH). The primary investigators had relationships in the work environment, District Heath Boards, tertiary settings, national ethical settings and the National Screening Unit setting. Once the topic had been identified, the primary investigator talked to Māori experts in Kaupapa Māori Research methodologies (by, for and with Māori), public health, Māori health and funding and planning. 

Kaupapa Māori Research methodologies allow exploration of Māori experiences and realities within a Māori worldview. In Aotearoa, kaupapa Māori researchers are in high demand and, therefore, time-poor. One way of addressing this was the establishment of a Māori governance group. While the primary investigator and other researchers ran the study, the Māori governance group had oversight at critical points. For example, when the proposal was being developed, developing information pamphlets and questionnaires and present at focus group hui (meetings). In line with Kaupapa Māori Research practice and the He Pikinga Waiora approach, Māori end users (people with lived experience) were involved in the design of the study. Involving Māori women began with hui facilitated by a Māori health literacy expert (SR). The outcome of the hui was the development of a Māori-specific pamphlet and initial discussions with Māori end users about the study design and purpose (co-production). 

District Health Board (DHB) Te Tiriti partners supported the project. Te Tiriti is the founding document of the colonial state of New Zealand, where health is recognised as a taonga (treasure) that should be protected. Te Tiriti reaffirmed Māori tino rangatiratanga (self-determination, sovereignty) and promised ōritetanga (equity) with British subjects. DHB partnership is with Iwi (tribe) of their rohe (area). A kaupapa Māori evaluation of the study (incorporated into this paper) was undertaken by our partner organisation (GM, a researcher at WaiResearch, the evaluation unit of Te Whānau o Waipareira). 

Māori ethics processes included throughout this project are consistent with the guidance in Te Ara Tika [23]. The project was informed by tikanga (correct procedure/practices) and values including aroha ki te tangata (respect for people), kanohi kitea (face-to-face contact), kōrero (looking, listening and then speaking), manaaki ki te tangata (care and reciprocity or to share and host people), kia tūpato (tread carefully), kaua e takahia te mana o te tangata (be cautious and/or do not trample the dignity of the people) and kaua e māhaki (do not flaunt your knowledge) [24,25,26]. The study incorporated whakawhanaungatanga (building relationships) and koha (reciprocity through kai (food) and the provision of vouchers to acknowledge participant time). 

### 2.2. Ethics: National, Local and Indigenous Māori Approvals

The feasibility study, the associated focus groups and evaluation received ethical and research office approval from the National Screening Unit Research and Evaluation Committee and the NCSP National Kaitiaki Group for the use of wāhine Māori data and review of the study manuscript. The studies also received approval from the Metro Auckland Clinical Governance Forum (primary care clinical leadership), individual Primary Health Organisation (PHO) and practices and Te Whānau o Waipareira research approval. The study was approved by the New Zealand Health and Disability Ethics Committee (HDEC) under reference number 16/STH/176. It was registered on the Australia and New Zealand Clinical Trials Registry under Intervention trial number: UTN U1111-1188-3226. We note that the participant with an early cancer diagnosis made as a result of this study gave her explicit permission for her case to be described and published.

### 2.3. Study Design and Participant Eligibility

We designed a single-arm feasibility and acceptability study, which was run for 12 months from December 2016 in West Auckland, Aotearoa. The main sample population was wāhine Māori aged 30–69 years, who were residents in Waitematā or Auckland District Health Board catchment areas in Aotearoa, and who had either never been screened or were under-screened (≥5 years since last screening event). West Auckland general practices were nominated by PHOs as having both a high proportion of Māori patients and low rates of cervical screening. Data matching between PHOs and the National Cervical Screening Programme (NCSP) Register provided lists of women from these practices who met the study criteria. Exclusion criteria were pregnancy, history of high-grade cytology or a benign total hysterectomy and any woman with symptoms suggestive of cervical disease. Any eligible women excluded from the study (e.g., pregnancy) but requiring follow-up were referred appropriately (colposcopy, primary care and/or independent service provider). Women were able to withdraw from the study at any time, for any reason, without impacting on medical care. 

### 2.4. Materials Development 

With the consent of investigators, we adapted several of the patient materials originally used in the Australian iPap study (exploring the acceptability of self-sampling in Australian urban and rural women who were non-respondents to the regular screening programme, aged 30–69) for an Aotearoa context [27]. This included changes appropriate to the health literacy demands placed on women and cultural appropriateness. It also included key messages addressing women’s concerns of not performing the test correctly and highlighting that oncogenic HPV testing is not a test of relationship fidelity. Draft study resources, including the HPV self-sample kit instructions with pictorial instructions, brochure and participant information sheet, were tested through an iterative process. Initially, with the first two focus groups of wāhine Māori, feedback was obtained on different options for the graphical design of the brochure and instructions. As a result of the feedback, the content, language and design of participant materials were amended. The amended documents (Supplementary Materials file S3) were tested with participants in the final round of focus groups.

### 2.5. Focus Groups

In addition to the main feasibility study, between December 2016 and October 2017, a total of seven focus groups were held with women from four practices who were also identified from PHO data-match lists as being ≥30 years old, under- or never-screened and of Māori, Pacific or Asian ethnicity. Focus groups were designed to test both the research materials (information sheet and instructions) and the study processes (consent, instructions, result management) and finally to review the amended materials. Women at the focus groups were also offered the opportunity to perform an HPV self-sample or to receive the usual care if they wished to participate in the main study. A koha was offered to women who attended the focus groups. The focus groups were co-facilitated by the research nurse and either a Māori health literacy consultant or a representative of the Asian or Pacific community.

### 2.6. Questionnaires

Two questionnaires were developed for women participating in the study. Firstly, women attending focus groups and participating in the main study were offered a questionnaire to assess their knowledge about HPV, cervical cancer and vaccination, based on an HPV public knowledge questionnaire from Patel et al. (2017), adapted for a New Zealand context [28] (Supplementary Materials file S1). This questionnaire was optional and was completed prior to any HPV self-sample testing in order to more accurately reflect baseline levels of women’s HPV knowledge before a detailed discussion took place related to self-sampling consent. All participating women were also asked to complete post-test questionnaires (Supplementary Materials file S2). These examined the barriers and enablers to routine cervical screening, including whether women found the self-sample acceptable and how they might prefer to be tested in the future. The questionnaire was localised from the Australian iPap study responder’s post-test questionnaire, with permission from the iPAp investigators [27,29].

### 2.7. Contactability and Invitation

A Māori cervical screening specialist nurse was appointed to work with participating practices to invite eligible women to attend a clinic and “do a new self-sample for cervical screening”. If women could not be reached initially, a total of five contact attempts were made, including phone calls at different times/days, texts and in some cases, a letter. Women who could be contacted and who agreed to participate booked an appointment to attend a study clinic in a community location. 

### 2.8. Clinic Consenting and HPV Testing

Women who agreed to participate attended a clinic for a 30 min consultation with the study nurse. In a private space, the research nurse explained kanohi ki te kanohi (face-to-face) the study and discussed the participant information sheet and consent form and answered any questions. Women were offered the knowledge questionnaire, then the research nurse explained how to take the self-sample using pictorial instructions and noted that there was a post-test questionnaire to go through afterwards. A study number was assigned, and consent and laboratory forms were prepared. Participants had the option of taking the test kit home (and returning it to the clinic within seven days) or performing the test in the clinic rooms (e.g., in the bathroom or private room). Women who completed the test at the clinic completed the post-test questionnaire at the same visit. Women who completed the test at home either completed the post-test questionnaire over the phone or in person at a follow-up meeting. 

Consented participants were provided with a self-test kit including a single blister-packed sterile flocked swap (511CS01 Copan, Italy) and the cobas PCR Media tube (Roche Molecular Systems, Pleasanton, CA, USA), along with test kit instructions and labels. 

All samples were tested for oncogenic HPV types using the cobas 4800 HPV assay (Roche Molecular Systems, Pleasanton, CA, USA) at IANZ accredited Auckland Anatomic Pathology Service. This assay specifically detects HPV types 16 and 18 and 12 other oncogenic HPV types concurrently as a group. The protocol for testing self-taken swabs on the cobas HPV test was based on the validation by the PAVDAG study [30]. Samples were tested within seven days, and results were reported to both the study nurse and the nominated primary caregiver with a copy to the NCSP register (with a research flag). 

### 2.9. Results Management and Follow-Up

Any invalid results were recorded, and a repeat test was offered to the participant. Negative results were provided to women even if the routine practice approach was not to provide negative results. Participating women were asked their preference for receiving this result (e.g., a letter or phone call). Negative results were communicated with advice to return for a routine cervical screen at the current NCSP recommended interval. HPV positive results were provided to women kanohi ki te kanohi (face-to-face) by the research nurse unless participants stated a preference otherwise. Women who were positive for HPV16 or 18 were referred directly to colposcopy. Women who tested positive for the group of 12 other oncogenic HPV types were triaged in the current standard process, i.e., with speculum examination and Liquid-based Cytology (LBC) by the study nurse. Women with any positive LBC results of ASC-US or worse were referred directly to colposcopy; women with negative LBC results were referred for management by their usual primary care/provider team for a repeat LBC test in 12 months, according to the NCSP standard of care. Extra support to attend a screening and/or colposcopy was provided by the study nurse (see Supplementary Materials file S6 for the flowchart of management of the algorithm).

Women’s participation in this feasibility study was recorded on the NCSP register (with a research flag) for additional safety and follow-up. Women were advised of this in the Participant Information Sheet (brochure), and this was also discussed verbally by the research nurse during the consenting process. Although test results were managed by the women’s usual care provider, our research nurse monitored positive results and provided a failsafe follow-up process. Our study committed to follow-up ≥90% of women who tested positive for HPV. In partnership with the colposcopy service, Independent Support Providers (ISPs) and community health workers, the study team attempted to provide all appropriate support (e.g., supported decision making over time, assistance with transport, child care, visit attendance support) to ensure that women with a positive HPV result were followed-up. There was no charge to women for support services. If women did not attend their colposcopy appointment, the research nurse was notified of the non-attendance and arranged the appropriate support for a follow-up. A study completion form was developed for women who did not attend and who were discharged back to their primary care provider; this was followed up by the research nurse.

## 3. Evaluation

WaiResearch (the research unit of social services organisation Te Whānau o Waipareira) conducted a process and short-term outcome evaluation of the study. The evaluation aimed to assess how well the feasibility study aligned with Kaupapa Māori Research best practices and best outcomes and to identify practices that contributed to an overall positive experience for women during their involvement in the study. As well as document review, interviews were conducted with 23 participants, two practice staff and two study nurses. The evaluation took a kaupapa Māori and a strength-based, participatory approach with a strong focus on national policy implications. 

## 4. Results

### 4.1. Data Matching, Contacting, and Recruiting Women

We recruited seven urban Auckland general practices to the main study who were each provided with PHO lists of their patients who met the eligibility criteria. Across all practices, a total of 366 potential participants with recent contact details were identified. The practices then provided contact information to the study nurse to begin recruiting. Initially, we were only able to contact 114 women, and this low rate persisted, despite conducting the planned five contact attempts at different times of day, using three methods (phone, letter, text) in different orders. We also utilised different staff to contact women, both Māori and non-Māori research nurses, kaiāwhina (Māori community health workers) and PHO-employed Māori nurses. Transience and frequently changed phone numbers were likely to be causative factors among the study population. Some of the women who were contacted were found to be ineligible [17], others declined to participate [25], while others who agreed did not attend subsequent clinic appointments [31] (Table 1). A total of 46 self-samples were tested for HPV from the clinic invitation group. 

From our separate focus groups, a further 38 eligible women (18 from the three Māori focus groups, 6 Chinese, 5 Indian, 9 Pacific) who attended also consented to participate in the study and provided a self-sample for HPV testing. This brought the total number of HPV tests in this study to 84. The age range of the 84 participants was 30–68 years, and the median age was 45 years.

### 4.2. Self-Sampling HPV Results and Follow-Up

Of the 84 women tested for oncogenic HPV, a total of 5 returned positive results (6%) consisting of one HPV18 and four non 16/18 HPV positive results. One participant received an invalid test result; however, the participant acknowledged that she intentionally did not perform the self-sample. The remaining 78 results were negative. All swabs were receipted at the laboratory within the validated 7-day period between sampling and laboratory registration and processing. 

All four non16/18 HPV positive participants had records showing their last screening event as being between 8–20 years prior, and they ranged in age between 35–60 years. One participant was subsequently discovered to have had a recent negative cytology test; her results were included in this analysis based on “intention to treat”. Two participants were followed up with a pap smear and had negative cytology within 2 weeks to 3 months. The fourth was initially reported as lost to follow-up but subsequently had a negative cytology result in primary care when she returned from overseas 6 months later. 

The participant positive for HPV 18 was referred to colposcopy where a stage 1B Adenocarcinoma was subsequently diagnosed on histology. 

### 4.3. Case Review- Stage 1B Adenocarcinoma

This participant is a wāhine Māori who had not been screened for 11 years but had a normal smear history prior to this. There was a delay (4 months) in providing her HPV self-sample results face-to-face due to appointment rescheduling, so she was managed within the study as a protocol deviation (time to results discussion was expected to be 2 weeks). The timeline from the provision of results to attendance at follow-up took a further 3 months due to an appointment non-attendance for colposcopy services and further delays due to participant rescheduling. Ongoing contact, rescheduling support and offers of support to services were provided throughout. 

At the colposcopy, abnormal cervical features suspicious for cervical cancer were noted, and cytology and biopsies were taken. The cytology reported abnormal glandular cells consistent with Adenocarcinoma, and the biopsies reported Adenocarcinoma in situ (AIS). A cold knife cone was recommended, and the treatment histology reported a stage 1B Adenocarcinoma. The participant was notified of this result and referred for a staging MRI and to the Gynaecology Oncology Service for treatment. She later received a radical hysterectomy which demonstrated no residual disease and negative pelvic lymph nodes. Follow-up has been normal, and the participant is clinically well. The study team kept up to date with the participant’s clinical progress via the study nurse. It was estimated that 40 study nurse hours were spent supporting this participant during her assessment and diagnosis. A formal case review with Gynaecology Oncology Services determined the initial delay in results provision did not significantly impact the participant’s treatment outcome. 

### 4.4. Following up on Non-16/18 HPV Positive Women

Following up on women who tested positive for other oncogenic types of HPV was time-intensive and took an average of 5 h of skilled nursing time per patient, including specific counselling to facilitate shared decision-making. The follow-up process would begin with a phone call to arrange a kanohi ki te kanohi visit, however, for all women this caused anxiety, and they wanted to be given their results at the time of the call rather than wait for an appointment. HPV test results were therefore discussed in-depth by phone and arrangements made for a follow-up cytology test. Having to attend a clinic appointment introduced a further barrier for women, and appointments were often deferred or negotiated around women’s work and whānau (family and community) commitments.

### 4.5. Feedback on Self-Sampling

Of the 84 women who completed a self-sample, 58 also answered post-test questionnaires (a response rate of 69% (see Supplementary Materials file S5)). Not all women answered all questions. Table 2 shows the ranked responses for not having had a cervical smear test recently. 

Although multiple responses were allowed, no respondents said they would prefer a nurse/doctor to take a usual smear test—all preferred self-sampling either at home (33/58) or a GP clinic (25/58). None responded that they did not intend to screen (either by smear or self-sample) again. The most popular preference for receiving a self-sample kit was for it to be posted (25/60), followed by picking it up from the GP clinic (17/60), while the remainder of responses were “I do not mind” (13/60), face-to-face delivery by a community health worker (4/60) or from a community location (1).

When asked to choose their top two reasons for preferring a self-sample, participants’ highest-ranked selections were: its simplicity (33/59) and being less embarrassing (26/59), followed by not requiring an appointment with a doctor or nurse (24/24) and the test does not require a speculum (18/24). 

Comparing the self-sample to their last smear test, all (50/50) respondents to this question said the self-sample was easier. Almost all said it was more convenient, less embarrassing and less uncomfortable. Three women said there was no difference in some of these factors. Twelve thought the self-sample was also more accurate; however, most (25/38) were unsure or did not know. None said the smear test was more accurate.

Almost all respondents (55/57) said it was easy to use the swab and did not feel embarrassed. Most were confident they had done it correctly. Most respondents (44/49) said taking the swab was not at all painful or uncomfortable, while eight women noted a little discomfort (see Table 3 below). All respondents (53/53) said they would recommend using the self-sample to a friend or whānau.

### 4.6. Focus Group Feedback on Materials 

There was positive feedback from wāhine Māori at the focus groups about the proposed title and stylised graphics on the brochure

*…when I saw that ‘He taonga he tapu’* [title of patient information brochure given by a Māori woman in the first focus group meaning precious, significant or important entities which are sacred and must be actively protected] *brochure, I knew there was something special about the approach to wāhine* (Māori women) 

There was a negative response to anatomically explicit pictures in the sampling instructions. 

Pacific women generally expressed a preference for warmer colours and pictures of real women, while women in the Asian focus groups preferred the name of the study to be informational and ‘state what it means’. There was a preference for very clear visual images on instructions.

The materials were amended as a result of this feedback. Differences between ethnic preferences were resolved by designing two brochures, accommodating wāhine Māori preferences separately. Content changes included addressing women’s concerns of not performing the test correctly and highlighting that HPV testing is not a ’test of relationship fidelity ’. Amendments were re-tested in the third focus group with very favourable feedback. 

See Supplementary Materials file S3 for the final brochures. Moreover, see Supplementary Materials files S4 and S5 for the detailed results from the HPV knowledge, barriers and self-sampling acceptability questionnaires.

### 4.7. End of Study Evaluation Findings–Interviews with Wāhine Māori

Twenty-three wāhine Māori were interviewed as part of the end of study evaluation work. They reported that they found their involvement in the self-sampling feasibility study culturally appropriate and empowering. 

Specific observations that may be useful for future work include:

Having a study nurse who was also wāhine Māori working with them was conducive to culturally competent care and enhanced the acceptability of HPV-SS.

Tautoko, or the support they received, was important for all the women spoken to. The study nurse helped the women make decisions they were comfortable with, and for many, this was integral to their decision to do the self-sample. 

Almost all of the women reported that they received their test results from the study nurse or Kaiawhina in a manner that reduced associated stress.

Transparency of purpose and process, kotahitanga (shared experiences and purpose) was also identified as a key success factor in getting initial buy-in from the women interviewed. 

## 5. Discussion

This feasibility study explored key elements of the HPV self-sampling process in never- and under-screened Māori, Pacific and Asian women: (1) co-creating the HPV self-sampling materials; (2) contacting and inviting eligible women to participate; (3) collecting survey data to understand the women’s experience of the existing screening process and self-sampling; and (4) result management and follow-up. This work contains policy-relevant information for the NCSP on the resources required to implement an effective self-sampling programme to improve equity in cervical cancer screening. We explore each of these elements in turn below.

We co-designed new patient information materials taking a health literacy and ethnic-specific approach. Feedback from the focus groups led to two brochures being designed to accommodate different ethnic preferences (Supplementary Materials file S3), and all of the wāhine Māori who took part at the end of study interviews reported that they found the invitation process to be socially and culturally appropriate. Our follow-up processes included shared decision-making principles [31,32,33] at both the screening and the diagnostic/treatment steps in the pathway. The women reported receiving appropriate information to build on their existing knowledge as well as support in using the self-sampling kit. Having a culturally-concordant wāhine Māori study nurse was important in enhancing the acceptability of the self-sampling process. These findings are consistent with previous research [22], which explored the potential acceptability of HPV self-sampling in wāhine Māori and reported that both women and health care professionals emphasised the importance of cultural competence and empathetic support. For Chinese women, language was also a notable barrier to engagement with screening, and linguistically appropriate materials need to be made more widely available.

Identifying and contacting priority group women overdue for a cervical screen was challenging and resource-intensive, with only a third of eligible women able to be contacted after up to five attempts and 12% of those taking a sample. Of those who made an appointment, nearly a third did not attend the primary care practice setting. There are known barriers to primary care access in Aotearoa, New Zealand [34,35,36,37]. The focus groups led to the highest uptake of self-sampling, and in discussions about their experience of the current screening programme, some Pacific women indicated that they would prefer to discuss and attend a screening in a group setting. In contrast, almost half the women who completed the survey said they would prefer a self-sample kit to be sent by post. Furthermore, previous research has demonstrated that a self-sample kit sent to screening non-attendees can significantly increase attendance relative to a screening reminder [38]. Further research has recently been completed showing that a postal self-sample kit increases engagement [39], but it is likely that multiple approaches are needed, potentially with tailoring by different ethnic groups, age groups and communities [40]. Any future programme will need to ensure multiple access and outreach processes are in place to ensure maximum uptake.

The most frequently cited reasons in our study for not having had a smear test recently were feeling embarrassed, a previous bad experience, fear of discomfort, lack of time and not feeling comfortable asking for a test. These factors were also reflected in the quotes provided during focus groups and interviews. The quotes across all ethnicities also underscored the importance of the relationship with the smear taker, with women preferring someone they could feel comfortable with, a female smear taker and someone from their own culture. By contrast, HPV self-sampling was highly acceptable, with all respondents indicating they would prefer self-sampling either at home or at a GP clinic to a clinician smear test. The most frequently cited reasons for preferring a self-sample were its simplicity, being less embarrassing, not requiring an appointment with a doctor or nurse or a speculum and that the test is free. Just under half the women indicated they would prefer a self-sample kit to be posted out, while the second-most preferred option was to collect it from the GP clinic.

These findings are consistent with previous research. In a recent review [38], common barriers to cervical screening included embarrassment and discomfort and practical challenges such as time and cost. They found that women across multiple studies prefer self-sampling over clinician collected samples due to reduced embarrassment, discomfort and time pressures. Previously, patients have raised concerns about whether self-sampling is as effective as clinician sampling in detecting HPV [41]. Interestingly, this did not emerge during the current research. For clarity, a meta-analysis has demonstrated that self-sampling results are comparable to clinician collected samples [38].

It is notable that wāhine Māori reported the current screening programme as being disempowering while their involvement with this feasibility study, by comparison, was empowering. In other international Indigenous experiences, Aboriginal women, who reported screening as being shameful, invasive and uncomfortable nonetheless, also perceived self-sampling as a way of exerting control over their own health and giving them a sense of empowerment [42]. It may be that exploring and promoting messages of empowerment or participation enablers such as referral vouchers for free service provision might increase uptake of self-sampling in wāhine Māori. Additionally, in their recent position statement, Hei Āhuru Mōwai (national Māori cancer leadership group) called for Māori governance of the NCSP and sufficient resourcing to develop a Māori-led communications strategy including HPV self-sampling guidelines and the autonomy to enrol women in the programme, monitor and evaluate its progress independently [43]. 

Five of the eighty-four under-screened women who took part in this study and conducted HPV self-sampling returned oncogenic HPV positive results. One of these women was subsequently diagnosed with an early-stage invasive Adenocarcinoma and underwent a radical hysterectomy. One of the study’s aims was to investigate what resources were required to support at least 90% of HPV positive women to attend follow-up testing or treatment. Of the five women who tested positive for HPV, one was later found to have recently had negative cytology, one was lost to follow-up and eventually had a later opportunistic screen which was negative, and one declined to attend colposcopy, then did not attend a subsequent appointment and finally attended a rescheduled appointment where she was diagnosed with Adenocarcinoma. The other two women attended colposcopy and had negative cytology. In total, and in addition to the resource-intensive nature of inviting women to take part in self-sampling, an average of 5 h was required at follow-up for each of the women who tested positive for other oncogenic types of HPV. Without such diligent follow-up, it is likely that the woman diagnosed with cancer may have presented at a later stage. 

Challenges with achieving follow-up in the diagnostic pathway are likely to account for an important proportion of cervical cancers in wāhine Māori and Pacific women. In a recent review of invasive cervical cancers in New Zealand, between 2013 and 2017, over half of the women diagnosed with cervical cancer had been screened in the previous seven years, with a quarter of those having a high-grade abnormal screening test in that time [44]. More effective follow-up could have prevented or reduced the severity of these diagnoses. As opposed to only 16% of European women, 40% of wāhine Māori and 53% of Pacific women screened in that time period, prior to the diagnostic episode, had had a high-grade cytology result. 

There are a relatively small number of studies that have focused on HPV self-sampling in different Indigenous populations (see [28] for a recent review), and few of these detail the follow-up processes. In the Australian iPap trial, 62.2% (N = 28) of those women who tested positive for HPV 16/18 attended colposcopy within 6 months [24]. Two women declined clinical investigation, and a further six women were sent reminder letters at 3 and 6 months but had not had any further investigation by the end of the trial. Recent research [45] has reported success in using a community-based service model that respects Aboriginal cultural approaches to recruit under-screened and never-screened Aboriginal women to complete the cervical cancer screening and support them to engage in follow-up where necessary. More ethnocentric work is urgently needed to understand how follow-up processes might be managed in a culturally appropriate way with “strategies [that] centre Indigenous leadership, knowledge, solutions and community” [46].

One of the many barriers to reducing the burden of cervical cancer in Indigenous communities is knowledge about screening and how it can lead to the prevention of cancer [46]. We started exploring women’s knowledge about HPV in our study, but it was seen as a barrier to participation in the rest of the study and undermined women’s confidence, and so we terminated its use. It is possible that a new questionnaire with different framing, format and tone would have overcome these problems. Future research could usefully explore knowledge about HPV in light of the forthcoming changes to the Aotearoa NCSP, but this should be decoupled from research using self-sample kits. Due to the nature of the research and our interest in exploring HPV self-sampling in never- and under-screened wāhine Māori, Asian and Pacific women, only a small number of women (84) were recruited to this study, and although they were eligible, we did not recruit any truly never-screened women. Consequently, the views of those women who we were unable to reach or who declined to take part are not represented. Furthermore, we focused on women from one urban region of Aotearoa. Women from rural communities may well have different views. However, this feasibility study has paved the way for a larger randomised controlled trial that sought to address some of these issues [39,47], and another study recently published was conducted in a rural population [20].

## 6. Conclusions

Cervical screening uptake and cervical cancer outcomes for wāhine Māori, Asian and Pacific women currently lag behind those for other groups in Aotearoa, especially European women. The forthcoming introduction of HPV primary testing into the NCSP allows the possibility of offering women HPV self-sampling as an alternative to clinician collected sampling. In this co-designed feasibility study, we found that HPV self-sampling was acceptable to under-screened urban wāhine Māori, Pacific and Asian ethnicity women in Aotearoa. It was also effective at detecting HPV and early cervical cancer in these populations. Further research is required to extend the findings to rural communities and to tackle challenges around identifying and contacting never-screened and under-screened women. However, HPV self-sampling, accompanied by information and processes that are women-centred and culturally appropriate, appears to be a more acceptable way of engaging with the screening programme for these under-screened women than clinician-collected samples.

This study provides policy-relevant information on self-sampling for when the Aotearoa NCSP transitions to primary HPV testing. Our findings, alongside the large volume of deferred screens due to the burden of COVID-19 testing on our clinics and laboratories, underscore the urgency of implementing HPV primary screening to enable much-needed self-sampling in Aotearoa.

## Figures and Tables

**Table 1:** Summary of eligibility, contactability and uptake of study offer amongst Māori, Pacific and Asian women in West Auckland, New Zealand.

Summary of Recruitment		
Category	Number of Women	Relative Percentage
Eligible women identified	366	
Able to be contacted (5 attempts)	114	31% of women initially identified as eligible
Excluded on contact	17	
Declined	25	22% of women contacted
Did not attend clinic appointments	31	35% of women who agreed to participate did not attend
Completed the self-sample after invitation to attend clinic	46	47% of women contacted (after exclusions)
Completed the self-sample at focus groups	38	93% of eligible women who attended
Total self-sample HPV tests conducted	84	

**Table 2:** Reasons for not having a smear test recently (or never), ranked highest to lowest.

Response Option (N = 58)	Number Selecting as Main or Contributory	Number for Whom it Was Main Reason
A test from a nurse or doctor is/would be embarrassing	27	16
I have had a bad experience in the past having a test	22	13
A test from a nurse or doctor is/would be too painful or uncomfortable	21	13
It is hard to find the time to have a test	17	9
I don’t/wouldn’t feel comfortable asking for a test from my nurse or doctor	17	4
It is hard to find the right nurse or doctor, or it is hard to get an appointment	12	3
I don’t think I need a test	12	2
I am not having sex	10	8
It is too expensive to have a test	10	1
My nurse or doctor has not suggested a test	9	2
I don’t know if or when I should have a test	9	2
I have not received a reminder letter to have a test	8	1
It is hard to travel to an appointment	7	2
I don’t think the test results are accurate enough	5	0
I have had a hysterectomy *	4	1
I have never had sex	3	0

* Note that if the cervix remains after some type of hysterectomy, screening is still recommended.

**Table 3:** Participant responses to using the self-swab.

	Not at All	A Little	Very Much
It was easy to use the swab	0/57	2/57	55/57
Taking the sample with the swab was painful	44/49	5/49	0/49
Taking the sample with the swab was uncomfortable	38/47	8/47	1/47
I felt embarrassed	48/49	1/49	0/49
It was convenient	0/52	0/52	52/52
I am confident I did it correctly	0/55	5/55	50/55

## Data Availability

All data generated or analysed during this study are included in this published article and its supplementary information files.

## Supplementary Materials

The following are available online at https://www.mdpi.com/article/10.3390/ijerph181910050/s1, Supplementary Materials file S1: Participant HPV Knowledge Questionnaire; Supplementary Materials file S2: Post-test Barriers and HPV Self-Sampling Acceptability Questionnaire; Supplementary Materials file S3:Brochure and patient information materials; Supplementary Materials file S4: HPV Knowledge Questionnaire Responses; Supplementary Materials file S5: Post-test Barriers and HPV Self-Sampling Acceptability Questionnaire Responses; Supplementary Materials file S6: Flowchart: Management of women during feasibility study.
