# Supplementary material for: Human Papillomavirus (HPV) Self-Sampling among Never-and Under-Screened Indigenous Māori, Pacific and Asian Women in Aotearoa New Zealand: A Feasibility Study
Source: Int J Environ Res Public Health. 2021 Sep 24;18(19):10050. doi: 10.3390/ijerph181910050 (PMC8507781; doi:10.3390/ijerph181910050)
Supplement: Supplementary file 1 [file ijerph-18-10050-s001.zip › HPV SS Feasibility WDHB Supp data Aug21 1245v3.pdf]

Supplementary Materials 1: Participant HPV Knowledge Questionnaire

A. Causes of cervical cancer

Please answer these questions in as much detail as you can.

1. Thinking of cervical cancer, what do you think is its main cause?

2. What other causes of cervical cancer, if any, are you aware of? Please write down as many as you can think of.

Please ensure you have answered these two questions before turning over. Do not return to these questions once you have turned over.

4. Did you know that women may be tested for HPV when they have a smear test?

Yes

No

5. Which of the following statements apply to you? Please tick all that apply.

I have had a smear test in the past 5 years.

I or someone I know has been diagnosed with cervical cancer.

I have had the HPV vaccination.

I was offered the HPV vaccination but did not have it.

I do not know if I have had the HPV vaccination or not.

I do not know anyone who has had or been offered the HPV vaccination.

Someone I know has had the HPV vaccination.
Please specify their relationship to you ………………………………………..……

Someone I know was offered the HPV vaccination but did not have it.
Please specify their relationship to you ....……………..………………….

Supplementary Materials 1: Participant HPV Knowledge Questionnaire

B. HPV

3. Have you heard of HPV?

Yes (Please go to question 3a)

No (Please go to question 4)

Don't know (Please go to question 4)

3a. What do the letters HPV stand for?

3b. What is HPV?

3c. How does someone contract HPV?

3d. What is the relationship, if any, between HPV and cervical cancer?

Please make sure you have answered all questions so far to the best of your knowledge before you go onto the next page. Do not return to these questions once you go to the next page.

3e. Do you think the HPV vaccine will prevent all cases of cervical cancer?

Yes

No

Don't know

3f. What else do you know about HPV?

Supplementary Materials 1: Participant HPV Knowledge Questionnaire

3g. Which of the following sources have you heard about HPV from? Please tick all that apply.

Information from school/college.

Newspaper/magazine.

Internet.

Television.

Doctor/nurse/other health professional.

Friends/family.

Other (please specify) ………………………………………………………………….

Supplementary Materials 2: Post-test Barriers and HPV Self-Sampling Acceptability Questionnaire

1. What are your main reasons for never having had a cervical screening test (*smear* or *pap* test), or for not having had a recent test?

Please choose one main reason, then any other reasons that apply.

	**Main reason (choose 1)**	**Other reasons**
1. I don't think I need a test		
2. I don't know if or when I should have a test		
3. I am not having sex		
4. I have never had sex		
5. I have had a hysterectomy		
6. A test from a nurse or doctor is/would be embarrassing		
7. A test from a nurse or doctor is/would be too painful or uncomfortable		
8. I have had a bad experience in the past having a test		
9. I don't/wouldn't feel comfortable asking for a test from my nurse or doctor		
10. My nurse or doctor has not suggested a test		
11. It is hard to find the time to have a test		
12. It is hard to find the right nurse or doctor, or it is hard get an appointment		
13. It is hard to travel to an appointment		
14. It is too expensive to have a test		
15. I have not received a reminder letter to have a test		
16. I don't think the test results are accurate enough		

2. How you were invited to take part in this research project?

	Choose only 1 option
1. Text	
2. Phone	
3. Letter	
4. Face to face	
5. Combination – please write down details	
6. Other – please write down details	

Supplementary Materials 2: Post-test Barriers and HPV Self-Sampling Acceptability Questionnaire

3. Thinking about your experience of using the <u>self-test kit</u>, please rate to what extent the following statements apply to you:

	Not at all	A little	Very much	Unsure/don't know
1. I thought the instructions were clear				
2. It was easy to use the swab				
3. Taking the sample with the swab was painful				
4. Taking the sample with the swab was uncomfortable to do				
5. I felt embarrassed				
6. It was convenient				
7. I am confident I did it correctly				

4. In the future, would you prefer to see a nurse/doctor for usual cervical screening (*smear* or *pap* test) or take your own self-test for cervical screening?

	Choose only 1 reason
1. I would prefer a nurse or doctor	
2. I would prefer to take my own sample (self-test) at home	
3. I would prefer to take my own sample (self-test) at a GP clinic	
4. I don't intend to screen again	
5. Don't know/can't say	

a. If you would <u>prefer to take your own sample</u>, please indicate your top 2 reasons for this:

	Top 2 reasons
1. The test is free	
2. The test did not require an appointment with a nurse or doctor	
3. The test is accurate	
4. The test is less embarrassing	
5. The test is simple to do	
6. The test does not require the use of instruments (eg. speculum)	

b. If you would <u>prefer to take your own sample</u>, how would you like to be invited?

	Choose only 1 option
7. Text	
8. Phone	
9. Letter	
10. Face to face	
11. Combination – please write down details	

12. Other – please write down details	

c. If you would prefer to take your own sample, how would you like to receive the kit when you were due or overdue to be screened?

	**Choose only 1 option**
13. Collect the kit from the GP clinic	
14. Collect the kit from a community location	
15. Were posted out a kit to your home address	
16. Someone (eg Community Health Worker) gave it to me face to face	
17. I don't really mind	
18. Other – please write down details	

5. How would you like to get the results of your HPV self-test?

Please choose one option

	**Negative test (No HPV)**	**Positive test (HPV)**
1. Text		
2. Phone		
3. Letter		
4. Face to face		
5. Combination – please write down details		
6. Other – please write down details		

6. If your result is positive you will be referred to either your nurse/doctor for a cervical screening test or to the hospital clinic for a colposcopy test to check the cells on your cervix. Does this change the way you feel about doing this test?

	**Choose only 1 option**
7. Yes	
8. No	
9. Unsure/Don't know	
10. Other – please write down details	

Supplementary Materials 2: Post-test Barriers and HPV Self-Sampling Acceptability Questionnaire

7. Do you have any religious or cultural beliefs about self-testing that would be helpful to share with us?

	Choose 1 option
11. Yes	
12. No	
13. Unsure/Don't know	
14. Other – please write down details	

8. Would you recommend using the self-test to a friend or whānau?

	Choose 1 option
15. Yes	
16. No	
17. Unsure/Don't know	
18. Other – please write down details	

Only answer the following question if you have had a cervical screening test in the past:

9. Thinking back to both your last test done by a nurse or doctor and the self-test you took just now, which of the methods was:

	Self-test	Usual cervical screening test	No difference between the 2 methods	Unsure/don't know
1. Easier				
2. More convenient				
3. Less embarrassing				
4. Less uncomfortable				
5. More accurate				

10. Are there any comments about the self-testing that you would like to make?

*****Note that Supplementary material 3: Final HPV study patient information brochure is a PDF in a separate file***

Supplementary Materials 4: HPV Knowledge Questionnaire Responses

Supplementary Data 4: HPV Knowledge questionnaire responses

Number of respondents = 21

**General HPV knowledge questions**	Response<br>N
What do you think is the main cause of cervical cancer?	
Don’t know/ no response	8
Sex/body contact	3
A virus, infection	2
Lifestyle, diet	2
Family history/genetics	6
What other causes of cervical cancer, if any, are you aware of? Please write down as many as you can think of	
Don’t know/ no response	15
Bad health eg smoking, alcohol	3
Urinary infections, STI	1
Trauma, environment	1
Medicines, contraceptive pill	1
Have you heard of HPV?	
Yes	1
No	17
Don’t know	3
What do the letters HPV stand for?	
Don’t know/blank	20
Human Papillomavirus	1
What is HPV?	
Don’t know	17
A virus	1
An infection	1
A disease	1
An injection for girls	1
How does someone contract HPV?	

Supplementary Materials 4: HPV Knowledge Questionnaire Responses

**General HPV knowledge questions**	Response<br><br>N
Don't know/blank	18
Genetics	1
Contact, sex	2
What is the relationship, if any, between HPV and cervical cancer?	
Don't know/blank	20
To do with the cervix	1
Do you think the HPV vaccine will prevent all cases of cervical cancer?	
Yes	3
No	0
Don't know	18
What else do you know about HPV?	
Blank/'nothing'	19
Kids get the vaccine at school	2
Which of the following sources have you heard about HPV from? Please tick all that apply	2
Information from school/college	0
Newspaper/magazine	1
Internet	0
Television	2
Doctor/nurse/health professional	1
Friends/family	1
None of the above	0
Other (please specify)	16
Did you know that women may be tested for HPV when they have a smear test?	
Yes	0
No	21
Which of the following statements apply to you? Please tick all that apply	
I have had a smear in last 5 yrs	3
I or someone I know has been diagnosed with cervical cancer	6

Supplementary Materials 4: HPV Knowledge Questionnaire Responses

**General HPV knowledge questions**	Response<br><br>N
I have had the HPV vaccination	0
I was offered the HPV vaccination but did not have it	0
I don't know if I have had the HPV vaccine	6
I do not know anyone who has had the or been offered the HPV vaccination	5
Someone I know has had the HPV vaccination (Specify relationship to you)	1 (daughter)
Someone I know was offered the HPV vaccination but did not have it (Specify relationship to you)	1
Blank	8

Knowledge about HPV

Early in the study, 21 of the wāhine Māori attending focus groups and participating in the main study completed a knowledge questionnaire which covered causes of cervical cancer, awareness of HPV and HPV vaccination (25% response rate).

Responses indicated a very low level of HPV-related knowledge (See Supplementary Materials 5). Only one of the women had heard of HPV. Two women correctly responded that HPV was a virus or infection. Most did not know the cause of cervical cancer, though five women gave a correct response (sex, a virus, infection). 'Genetics' was given as a main cause by some (6 participants). None of the respondents knew that women may be tested for HPV when they have a smear.

Asked about other knowledge of HPV, two were able to identify that children received the HPV vaccine at school. Six did not know if they themselves had received the HPV vaccine. Six women said they knew someone who had had cervical cancer.

The offer of the knowledge questionnaire during recruitment and consenting was found to cause distress to some women, when administered *"those questions make me feel dumb – I thought this was about me doing my own test"*. Some women chose not to participate as they didn't know the answers. The questionnaire was therefore felt to be a barrier to women choosing to participate in the study and its use was discontinued after the focus groups, early in the study.

Supplementary Materials 5: Post-test Barriers and HPV Self-Sampling Acceptability Questionnaire Responses

Supplementary Data 5: Barriers to Cervical Screening Questionnaire

Number of respondents = 58, not all participants answered all questions

1. What are your main reasons for never having had a cervical screening test (*smear* or *pap* test), or for not having had a recent test?

Please choose one main reason, then any other reasons that apply.

	Main (N)	Other (N)
1. I don’t think I need a test	2	10
2. I don’t know if or when I should have a test	2	7
3. I am not having sex	8	2
4. I have never had sex		3
5. I have had a hysterectomy	1	3
6. A test from a nurse or doctor is/would be embarrassing	16	11
7. A test from a nurse or doctor is/would be too painful or uncomfortable	13	8
8. I have had a bad experience in the past having a test	13	9
9. I don’t/wouldn’t feel comfortable asking for a test from my nurse or doctor	4	13
10. My nurse or doctor has not suggested a test	2	7
11. It is hard to find the time to have a test	9	8
12. It is hard to find the right nurse or doctor, or it is hard get an appointment	3	9
13. It is hard to travel to an appointment	2	5
14. It is too expensive to have a test	1	9
15. I have not received a reminder letter to have a test	1	7
16. I don’t think the test results are accurate enough	0	5

Participant comments

- *I am tetaplegic and it is physically impossible to do at Drs (no hoist to get me onto bed)*
- *Uncomfortable with cubicle - no sense of real privacy to have it done*
- *none of these reasons*
- *I don’t like tests*

Supplementary Materials 5: Post-test Barriers and HPV Self-Sampling Acceptability Questionnaire Responses

- *there needs to be emphasis on the reasons why women need to have a smear test*

2. How were you invited to take part in this research project?

Invitation Method	N
Txt	10
Phone	40
Letter	6
Face to face	1
Combination please write down	2 (Ph,txt);
Other please write down	0

3. Thinking about your experience of using the self-test kit, please rate to what extent the following statements apply to you:

	Not at all (N)	A little (N)	Very much (N)	Unsure/don't know (N)
1. I thought the instructions were clear	0	0	57/57	0
2. It was easy to use the swab	0	2/57	55/57	0
3. Taking the sample with the swab was painful	44/49	5/49	0	0
4. Taking the sample with the swab was uncomfortable to do	38/47	8/47	1/47	0
5. I felt embarrassed	48/49	1	0	0
6. It was convenient	0	0	52/52	
7. I am confident I did it correctly	0	5	50	0

Supplementary Materials 5: Post-test Barriers and HPV Self-Sampling Acceptability Questionnaire Responses

4. In the future, would you prefer to see a nurse/doctor for usual cervical screening (*smear* or *pap* test) or take your own self-test for cervical screening?

	N
I would prefer a nurse or doctor	0
I would prefer to take my own sample (self-test) at home	33<br>(One said with a nurse)
I would prefer to take my own sample (self-test) at a GP clinic	25
I don't intend to screen again	0
Don't know/can't say	0

a. If you would <u>prefer to take your own sample</u>, please indicate your top 2 reasons for this:

	Top 2 reasons (N)
The test is free	14
The test did not require an appointment with a nurse or doctor	24
The test is accurate	3
The test is less embarrassing	26
The test is simple to do	33
The test does not require the use of instruments (eg. speculum)	18

b. If you would <u>prefer to take your own sample</u>, how would you like to be invited?

	N
Text	25
Phone	26
Letter	7
Face to face	1
Combination – please write down details	5 (phone, text or letter)
Other – please write down details	0

c. If you would <u>prefer to take your own sample</u>, how would you like to receive the kit when you were due or overdue to be screened?

	N
Collect the kit from the GP clinic	17
Collect the kit from a community location	1
Were posted out a kit to your home address	25
Someone (eg Community Health Worker) gave it to me face to face	4
I don't really mind	13
Other – please write down details	0

Supplementary Materials 5: Post-test Barriers and HPV Self-Sampling Acceptability Questionnaire Responses

5. How would you like to get the results of your HPV self-test?

	HPV Negative (N)	HPV Positive (N)
Text	27	7
Phone	22	19
Letter	6	4
Face to face	2	14
Combination please write down details	6	6
Other – please write down details; email	0	1= text to say please phone for result<br>1= phone if unanswered, text asking to be called back asap

6. If your result is positive you will be referred to either your nurse/doctor for a cervical screening test or to the hospital clinic for a colposcopy test to check the cells on your cervix. Does this change the way you feel about doing this test?

	N
Yes	4
No	45
Unsure/Don't know	7
Other – please write down details	1= if something is wrong I want to get it checked<br>1= if I know something is wrong I can do something about it

7. Do you have any religious or cultural beliefs about self-testing that would be helpful to share with us?

N=55	N
Yes	1
No	53
Unsure/Don't know	0
Other – please write down details	1= 'personal one on one'_

8. Would you recommend using the self-test to a friend or whānau?

N=53	N
Yes	53
No	0
Unsure/Don't know	0
Other – please write down details<br>Additional notes: "definitely"	0

Supplementary Materials 5: Post-test Barriers and HPV Self-Sampling Acceptability Questionnaire Responses

Only answer the following question if you have had a cervical screening test in the past:

9. Thinking back to both your last test done by a nurse or doctor and the self-test you took just now, which of the methods was:

N=50	Self-test	Usual cervical screening test	No difference between the 2 methods	Unsure/don't know
Easier	50	0	0	0
More convenient	40	0	2	1
Less embarrassing	41	0	1	0
Less uncomfortable	41	0	2	0
More accurate	12	0	1	25

10. Are there any comments about the self-testing that you would like to make?

Easy, less embarrassing, lovely lead nurse (Lucina), to make it simple and less scary

Easy to do, comfortable, non-intrusive

Self-testing removes embarrassment factor and is easy and convenient

I'm so happy I came and am part of this study

(the study nurse) was very encouraging and informative and I felt so relaxed and comfortable

Fast and carefree

Easy, good, quick. No pain whatsoever.

Very painless. Happy with the process.

Glad that I participated, was not as bad as I thought it would be

Easy and great idea!

I would recommend all women should know about the cervical smear to use a self sample test as it is less embarrassing

Supplementary Materials 5: Post-test Barriers and HPV Self-Sampling Acceptability Questionnaire Responses

Pretty easy
Found it quick, convenient, less embarrassing.
Great to have self-tests for the cervical screening or colposcopy. I am not sure if I would carry onto these tests if I was positive and these tests are too invasive.
The ease of self testing was great. In and out simple.
Love the idea of self-testing. My health is in my hands literally.
Prefer the self test
Thoroughly enjoyed it
would recommend self testing to everyone
like to see it implemented immediately and available to everyone
thank you for the opportunity to check myself without the embarrassment
love the idea, hope it becomes part of the normal for all females -so glad you asked me
I think this is a fantastic idea to get more females to get checked
my 8 sisters should do this test. Our Mum died of cervical cancer. It was too late
I believe this test would be of use for all women as it would increase use participation and also take away difficulty of lying on a bed to do a standard smear
Great idea - this would help me keep up to date
Very approachable nurse, very informative

Supplementary Materials 5: Post-test Barriers and HPV Self-Sampling Acceptability Questionnaire Responses

Women's current experience of screening

Focus group discussions and responses to questionnaires and interviews identified a range of personal, practical and health system barriers to participating in the current screening programme. Many of the women reported that they had made a conscious decision in the past not to participate in cervical screening. Specific findings by ethnicity, along with illustrative quotes are provided below.

Wāhine Māori

Wāhine Māori spoke of long periods or years since their last cervical smear, with most having made a decision not to have smears based on factors other than risk of cervical cancer. They talked of the invasiveness of smear testing and previous poor experiences (their own and whānau), creating a sense of vulnerability and disempowerment.

I had my first smear after I had my girl, I hated it then they said I had to come back a year later even though nothing was wrong! I came back I was 21 or 22 and I had an abnormal result they wanted me to come back again – that was it ….I decided I wasn't doing this!

Some women didn't consider screening to be necessary without symptoms or had not been provided with information about why it was important.

I feel well, when I think something is wrong then I will see the Doctor
… they (Pakeha [white New Zealander]*) will do it cause they are told, we need to know why but too many doctors and nurses just assume we know …. there is no conversation about it they just say 'you need to have one'.*

Some alluded to lack of a relationship of trust and caring with the smear taker:
…to feel comfortable with the person doing it and that they care (seem to care at least) would make it easier.

Chinese women

Language emerged as a key barrier to participation for Chinese women. Women talked of low awareness of cervical screening or the consequence of not doing smear tests. None had seen the Chinese cervical screening brochure or other translated information. Those who had been screened in New Zealand said the GP hadn't given enough information and/or explanation about the test:

I needed to be more prepared for what would happen
…you don't get a good explanation of the procedure and purpose
…GP just texts no information, no explanation
...I would like to be sent a brochure
All preferred support from Chinese healthcare providers, at least the first time.
Other barriers identified in the focus group for Chinese women were: discomfort; embarrassment; availability of a female smear-taker; 'bad comments' from others…. '*my colleague said it was scary so I didn't go*'; anxiety from *'not knowing what might happe*n' in the test; inconvenience: '*long waiting time at the clinic*' and cost: '*Price is not transparent - get charged after it's done'.*

Indian women

All five women at the focus group for Indian women had previously had cervical smears but had made a clear choice not to continue to participate in cervical screening. The key barrier was the test itself, which women described as painful, invasive and unpleasant:
…they tell us to relax and it's so painful and uncomfortable
Factors to do with the smear taker were important to some, such as lack of rapport and difficulties getting a female smear taker:
…there's always someone else there we get a different person each time
…prefer a mature person who can put you at ease.
Convenience factors were also mentioned such as the wait time and clinic hours:
…clinics are only open when I'm at work
…if it was easy maybe I would do it.

Pacific women

In both Pacific groups, embarrassment / shyness emerged as a main barrier to cervical screening:
..exposing my private parts for someone to look at

Supplementary Materials 5: Post-test Barriers and HPV Self-Sampling Acceptability Questionnaire Responses

…must be a female nurse who does it

…'tapu [taboo/sacred] part'…

Other barriers were discomfort, pain, time, cost, and previous bad experiences:

…I had a terrible experience at age 23 really really painful, I refused after that, but now I'm in my 40's I need to know if anything has happened

…I'm scared of the process you know, knowing how it's done

…very painful – felt like they were slaughtering me and so from then on I said NO!

Preference was expressed for discussing and attending screening in a group:

If you just give out pamphlets maybe we don't understand but here in a group I already feel more comfortable

Go to the churches - talk to the women doing activities then we can talk about smears
