# Supplementary material for: Human Papillomavirus (HPV) Self-Sampling among Never-and Under-Screened Indigenous Māori, Pacific and Asian Women in Aotearoa New Zealand: A Feasibility Study
Source: Int J Environ Res Public Health. 2021 Sep 24;18(19):10050. doi: 10.3390/ijerph181910050 (PMC8507781; doi:10.3390/ijerph181910050)
Supplement: Supplementary file 1 [file ijerph-18-10050-s001.zip › Supp 3 HPV Feasibility WDHB A5 Brochure.pdf]

Who is doing this study ?

Waitemata and Auckland District Health Boards are working with local clinics and Te Whānau o Waipareira to do this study.

Withdrawing from this study

Being part of this study is your choice. You can choose not to take part, or to withdraw from the study at any time. Your care won't be affected in any way. If you withdraw from this study, we will keep the information we have collected to the date you withdraw. We will not collect any new information after that.

Privacy and confidentiality

All information collected from you as part of this study will be confidential. You will be given a study number so that your name will not be used on the study documents. The study team will see the information and your test results. Your test results will be shared with your usual nurse or doctor, to make sure you get the correct follow-up. Your test results will also be held on the national cervical screening register. The information from this study will be marked as research on the register. Only approved cervical screening programme staff and health professionals will be able to get access to the research information.

National Cervical Screening Programme (NCSP)

A self-test is currently not offered to all women. It is only being offered to wāhine as part of this study. If at any time you wish to opt out of self-testing and have a standard test, talk to the study nurse or your own doctor or nurse.

All wāhine who participate in this study will be invited back for another cervical screen when they are next due for a test. The NCSP advises all women who have unusual bleeding, pelvic pain or discharge to see their doctor and not wait for their next cervical screen.

Contacting you again

We would like to contact some wāhine after the self-test to ask some more questions about your experience. You can choose whether you talk to us again or not. Your care will not be affected if you do not wish to talk to us again.

ACC statement

It is not likely that you will get injured in this study. If you did, you would be able to get compensation from ACC just the same as if you were injured in an accident at work or at home. You will have to put in a claim to ACC, which may take some time to be assessed. If your claim is accepted, you will receive funding to help you recover.

Further information

Thank you for thinking about being part of this study. If you have any further questions, you can contact the person who is leading this study:

Dr Karen Bartholomew, Public Health Physician, Waitemata DHB and Auckland DHB

If you have any other questions or complaints about the study, you can contact the Director of Wai Research at Te Whānau O Waipareira:

Dr Tanya Allport

For cultural support, please contact He Kamaka Waiora.

This study has received ethics approval 16/STH/176

AUCKLAND
DISTRICT HEALTH BOARD
Te Toka Tumai

Waitemata
District Health Board
Best Care for Everyone

He taonga, he tapu

Study into how acceptable HPV cervical screening self-testing is for wāhine Māori.

Information for wāhine

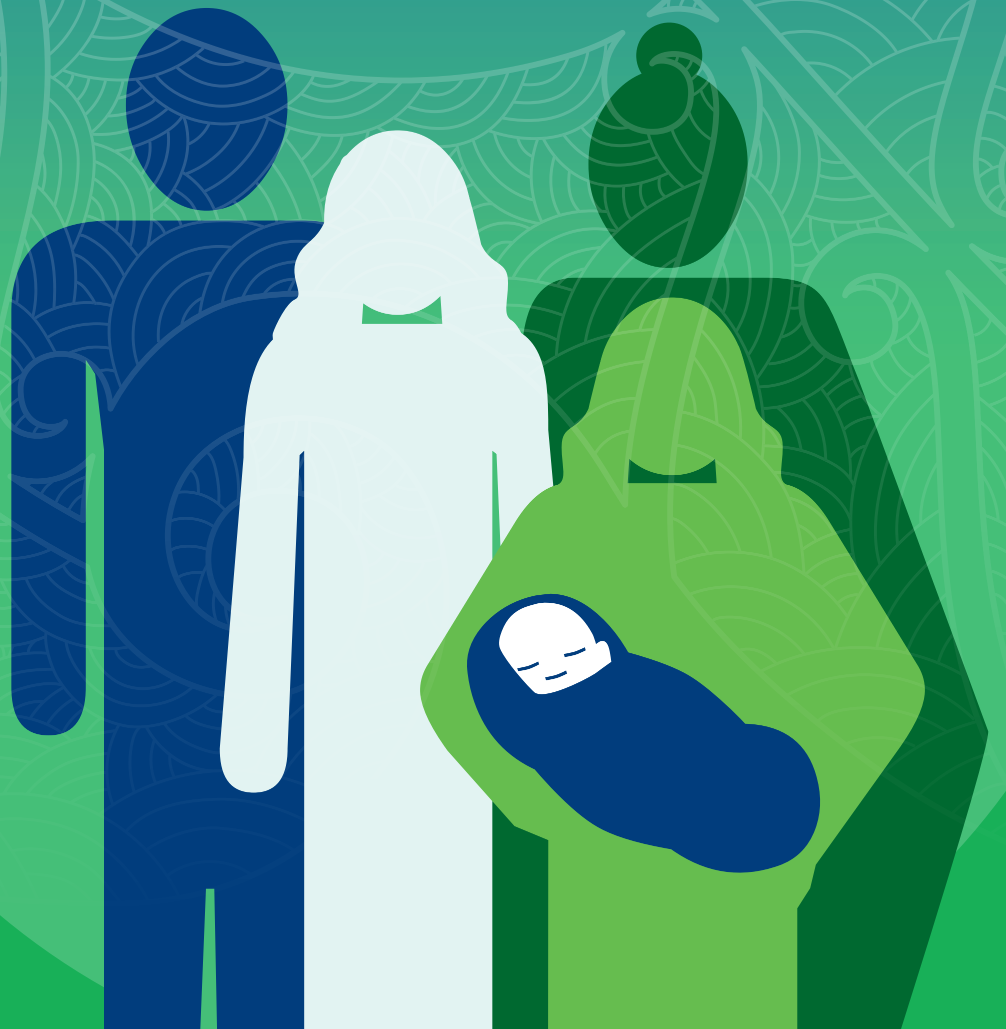

What is this study about?

The study seeks to find out if wāhine like using a free new test for cervical screening. In the new test wāhine use a swab (ūkui) in their vagina to find out if they have the human papilloma virus (HPV). HPV is a virus spread by skin-to-skin contact and is linked to cervical cancer. The new test is different from the usual smear test which a nurse or doctor does. The study will help the Ministry of Health decide how wāhine might have HPV tests in the future.

What am I being asked to do?

We would like to invite wāhine Māori in West Auckland to take part in the study.

1. We will ask you to come into your usual health clinic. The study nurse will explain what the study is about and answer your questions. If you decide to take part in the study you will need to sign a consent form.
2. You will then do the test using the kit and instructions sheet. You can do the test in the bathroom at the clinic, or at home and drop the test back into your clinic. Your test will be sent to a laboratory where they will test for HPV.
3. After you have done the test the study nurse will ask you some questions.

How will I find out my test results?

The study nurse or your nurse or doctor will contact you with your results within 10 days after your test. If your test results show you do not have HPV, you don't have to do anything else. Your own nurse or doctor will let you know later when you are due for your next smear test.

What if my test results show I have HPV?

We will ask you to come into the clinic to talk about what the results mean. We strongly recommend that you have a follow-up test to look for any cell changes that might need treatment. In this follow-up test a nurse or doctor at a GP or hospital clinic looks at your cervix, either to take a smear or do a colposcopy. There will be no charge for any follow-up tests. The study nurse can help if you would like support to get to the clinic, or if you want to talk more about what the test results mean.

It is your decision whether you have follow-up tests or not. We will talk to you about the follow-up tests and answer all your questions. We can also talk to your whānau if you want us to.

A positive HPV test does NOT mean you have cervical cancer.

More information

On this page there is more information about HPV, cervical cancer, reducing your risk of cervical cancer and the HPV test.

More about HPV

Being infected with HPV is very common. Four out of five men and women will have HPV at some time in their lives.

Some types of HPV stay in the body for a long time. For most women, having HPV does not cause any problems. Your body gets rid of the virus by itself.

Having HPV doesn't mean that your partner is being unfaithful to you. You could still have HPV even if you are in a long-term relationship with one person, are not currently having sex, or have not had sex in a long time.

More about how HPV is linked to cervical cancer

Certain types of HPV stay in the body for a very long time and can cause cell changes in the cervix that can lead to cervical cancer.

The most common types of HPV that cause cell changes that lead to cervical cancer are called HPV16 and HPV18. There are another twelve types of HPV that can cause cell changes that can lead to cervical cancer.

Following up a positive HPV test so we can check your cells, means we can pick up changes and treat them early.

Other types of HPV can cause minor changes to the cells of the cervix, or sometimes genital warts. These types are usually cleared by your body within one to two years and do not cause cervical cancer.

How can I reduce my risk of cervical cancer?

The best ways to reduce your risk of cervical cancer is to have the HPV vaccine which is free for males and females aged 9 to 26 years **and** have regular smear tests with your nurse or doctor.

How is HPV treated?

There are treatments for cell changes to your cervix caused by HPV. This is why it is important for you to have follow-up tests if your test results show you have the type of HPV that can cause cervical cancer. Treatment happens at a GP or hospital clinic and is very successful at stopping serious cell changes from becoming cervical cancer.

More about the HPV test

In this study you do the HPV test yourself. This is a very accurate test to check for HPV. You don't need to know where your cervix is to do this test. Testing yourself for HPV is only available in New Zealand, as part of this study. Some HPV tests are currently available with a cervical smear taken by a doctor or nurse. This test does **not** check for sexually transmitted infections (STIs) or HIV.

Is doing the HPV test myself right for me?

Some wāhine may find doing the test themselves easier than getting a smear with their nurse or doctor.

If you have had a hysterectomy, ask the study nurse whether self-testing is right for you.

If you have had the HPV vaccine, you can still take part in the study.

Are there any possible risks with self-testing?

In New Zealand at the moment we advise you not to do this test if you are pregnant. However overseas, pregnant women have done this test themselves and have not reported any problems.
