# Supplementary material for: Human Papillomavirus (HPV) Self-Sampling among Never-and Under-Screened Indigenous Māori, Pacific and Asian Women in Aotearoa New Zealand: A Feasibility Study
Source: Int J Environ Res Public Health. 2021 Sep 24;18(19):10050. doi: 10.3390/ijerph181910050 (PMC8507781; doi:10.3390/ijerph181910050)

Supplementary File 6: Flowchart
Management of women during the HPV self-sampling feasibility study.
From Bromhead et al., Human papillomavirus (HPV) self-sampling among never- and under-screened Indigenous Māori, Pacific and Asian women in Aotearoa New Zealand: A Feasibility Study.

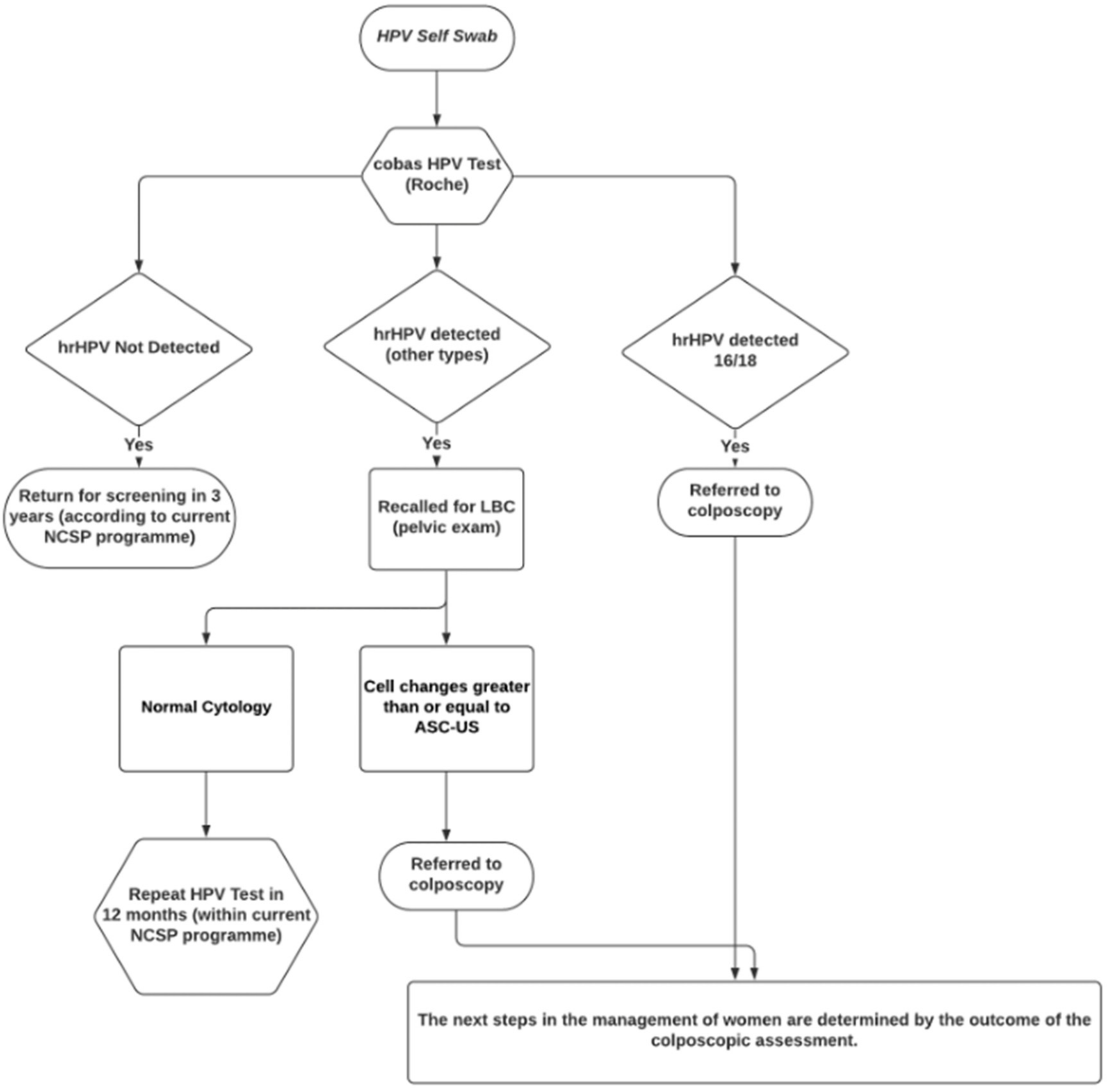

Supplement: Supplementary file 1 [file ijerph-18-10050-s001.zip › Supp 6 Figure 1 HPV Feasibility WDHB.pdf]
